# Recent Advances in Wearable Sensing Technologies

**DOI:** 10.3390/s21206828

**Published:** 2021-10-14

**Authors:** Alfredo J. Perez, Sherali Zeadally

**Affiliations:** 1 TSYS School of Computer Science, Columbus State University, Columbus, GA 31909, USA; 2 College of Communication and Information, University of Kentucky, Lexington, KY 40506, USA

**Keywords:** wearables, smartphones, sensing, fitness, mobile payments, financial technology, m-health, crowdsensing, Internet of Things, security, privacy, energy, COVID-19, SARS-CoV-2

## Abstract

Wearable sensing technologies are having a worldwide impact on the creation of novel business opportunities and application services that are benefiting the common citizen. By using these technologies, people have transformed the way they live, interact with each other and their surroundings, their daily routines, and how they monitor their health conditions. We review recent advances in the area of wearable sensing technologies, focusing on aspects such as sensor technologies, communication infrastructures, service infrastructures, security, and privacy. We also review the use of consumer wearables during the coronavirus disease 19 (COVID-19) pandemic caused by the severe acute respiratory syndrome coronavirus 2 (SARS-CoV-2), and we discuss open challenges that must be addressed to further improve the efficacy of wearable sensing systems in the future.

## 1. Introduction

**Figure 1:**
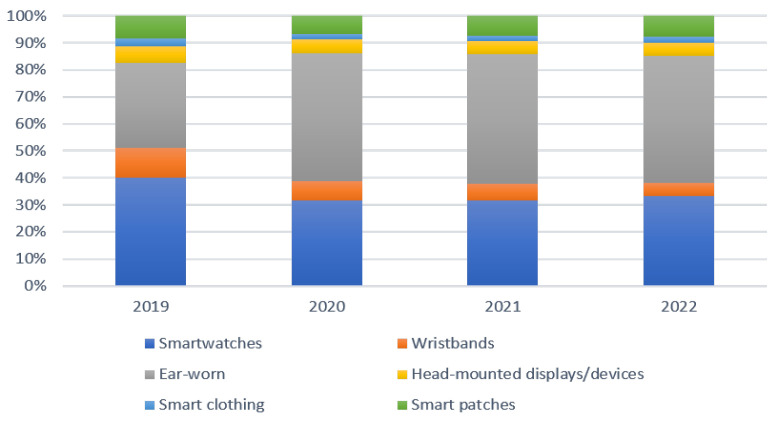
Consumer wearables device market share (2019–2022).

Wearable sensing technologies continue to improve rapidly with advances in sensors, communication technologies, and artificial intelligence (AI) in the past decade. Research and development in wearable sensing technologies are fueling a revolution in the creation of novel services in gaming, fitness, entertainment, and specialized applications in industries such as healthcare, security, and defense, among others. In 2020, the market for wearable devices was USD 80 billion, which has tripled in terms of annual revenue since 2014 and it is expected to reach USD138 billion by 2025 [1]. In the consumer wearables market, in 2019, smartwatches and wristbands dominated the market with a combined market share of 51%; as of 2021, the leading wearables are ear-worn wearables with a market share of 48%, followed by a 37% combined market share of smartwatches and wristbands [2]. Figure 1 presents the consumer wearable devices’ market share by device type (2019–2022).

Ear-worn wearables, in special hearables such as true wireless stereo (TWS) wearables, have surged from almost zero market share to a significant share of the wearable device market [1] since the introduction of Apple AirPods in 2016, and have significantly increased during the coronavirus disease (COVID-19) pandemic [3], caused by severe acute respiratory syndrome coronavirus 2 (SARS-CoV-2), as many people have worked and studied from their homes worldwide. During the pandemic, smart reusable masks that can detect SARS-CoV-2 and self-sterilize have become an active area of research and development [4,5,6,7,8]. The COVID-19 pandemic has also positively impacted the adoption of other consumer wearable technologies for mobile payment systems, patient tracking, contact tracing, and remote patient monitoring and treatment [9,10,11,12]. Combined fitness/medical-connected services was the leading market for wearable sensing technologies as of 2020 [13,14,15]. Other markets such as industrial wearables services, entertainment/gaming (i.e., augmented reality (AR) games and devices), and wearables for defense and security are also surging in popularity with recent technological advances in wearable technologies.

**Figure 2:**
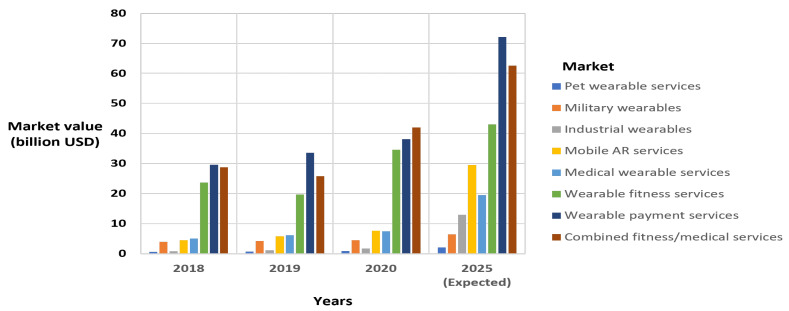
Wearable services market value.

According to latest market research analysis, by 2025, the wearable payments services market (around USD 72 billion by 2025) is expected to be larger than the combined fitness/medical wearables services market by approximately USD10 billion [13,14,15]. The wearable payments market has grown due to the adoption of near-field communication (NFC) in smartphones by manufacturers supporting financial payment standards [16,17,18], and the incorporation in the near future of NFC in new generations of smartwatches, fitness trackers, and other wearables such as smart rings [19]. However, by 2028, it is forecasted that the wearable fitness market will be approximately USD 138.7 billion [20], while the wearable payment services will remain around USD 80 billion [21]. Figure 2 presents the market value of wearable services for the years 2018–2020, and a projection for 2025 based on available market research data [13,14,15,22].

Consumer wearable sensing systems were initially researched with cellphones and smartphones during the second half of the 2000s. During that time, the widespread adoption of cellular communication in the world, the mobile Internet, and the embedding of sensors in cellphones such as location sensors, accelerometers, and cameras paved the way to the development of sensing applications (in particular applications related to human-centric activities) in urban environments at a low cost compared with the deployment of static wireless sensor networks (WSNs) to achieve the same human-centric sensing goals [23]. The research in this area led to the development of many applications in the context of participatory and crowdsensing systems [24,25] using not only embedded cellphone sensors, but using external sensors connected via Bluetooth. Table 1 presents a summary of related works in mobile and wearable sensing during the past decade.

Most of the works cited in Table 1 addressed specific aspects of mobile and wearable sensing systems, with many works focusing on smartphone-based sensing/crowdsensing systems in the past decade. In this work, we present a comprehensive review to provide the reader with not only a summary of past works but also new opportunities in wearables. Moreover, the unexpected COVID-19 pandemic has brought to the spotlight the use of wearable sensing technologies, and has positively shifted the perception and adoption of wearable technologies despite their privacy and security issues. Thus, while recent advancements in wearable sensing technologies have paved the way for the emergence of a plethora of services we are currently using in our lives, there are several areas still in need of further research. In this work, we describe current advances in wearable sensing technologies and services, and their use and opportunities to continue moving the field forward. The main contributions of this paper are as follows:

- We present a comprehensive review of current advances in wearable sensing technologies;
- We describe recent developments in communication, services, security, and privacy technologies for wearables;
- We discuss some research opportunities and challenges that we need to address in the future for wearable sensing technologies.

**Figure 3:**
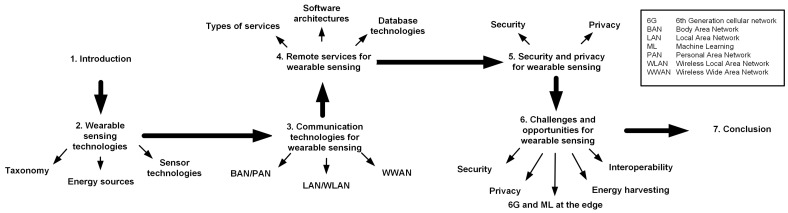
Paper organization.

We organize the rest of the paper as follows: In Section 2, we review the hardware architecture of wearable sensing devices. Section 3 presents communication technologies for wearable sensing. In Section 4, we discuss remote services for wearable sensing. Section 5 reviews security and privacy challenges and solutions for wearable sensing devices. In Section 6, we present challenges and opportunities in wearable sensing. Finally, Section 7 presents concluding remarks. Figure 3 presents the organization of this work.

## 2. Wearable Sensing Technologies

A wearable sensing device is a device that consists of sensors, actuators/output devices, a power generating unit, and an embedded computer, which may be implanted, worn, or carried around by a user [29,38]. This user may be a person or, in the case of some wearables, worn by animals. Depending on the characteristics and sensing goals of a wearable sensing device, it may be connected to external systems using the Internet either through a cellular network and/or wireless local area network (WLAN). External systems can store and conduct analysis using artificial intelligence (AI) techniques and may provide feedback to the user of the device. While the ubiquity of wireless sensing technologies has dramatically increased in recent years, early utilization of wearable sensing devices dates back decades ago [29,48]. As of 2021, there are at least 266 companies producing at least 430 wearable sensing devices [49] that can be categorized in a taxonomy based on three layers that include: market type, intrusiveness, and body location.

The first layer (market type) determines the ease with which a general user can typically access a wearable sensor device. Based on the market, devices can be grouped into consumer wearable sensing devices/systems and specialized wearables. Consumer wearable sensing devices can be further categorized into fitness, entertainment/gaming, security, or pet use [42,50]. Specialized wearables can only be acquired through specialized vendors, and they comply with special standards or may be regulated by laws that specify who may acquire and/or use them and the specific purposes for which each is designed. Thus, we can categorize specialized wearable sensing devices into industrial, healthcare/medical, security/defense, and research fields.

**Figure 4:**
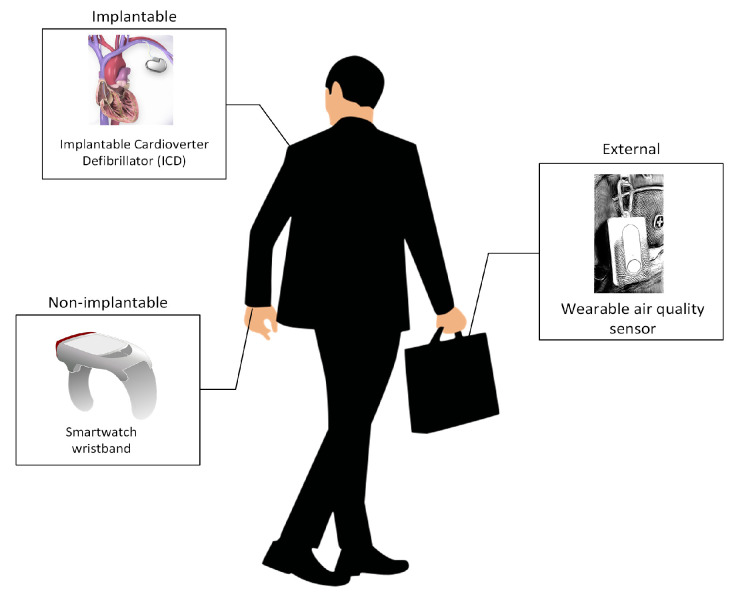
Wearable sensors based on intrusiveness level.

The second layer (intrusiveness level) determines whether the wearable can be implanted/placed into the body of a living organism (implantable), placed on/worn by a living organism (non-implantable), or carried by a user, for example, on a backpack (external). Under this classification, ingestibles would be classified as an implantable device [51]. The difference between a nonimplantable and an external wearable is whether the device is directly in contact with the body of the user (nonimplantable) or not (external). Figure 4 illustrates sensors based on intrusiveness level.

The third layer (body location) determines the placement of the wearable sensing device, which can be the head, trunk, arm, or leg. It is worth noting that these are general positions on the body of a user, so when we refer to the head, this location may include the neck, ears, or eyes. Thus, an example is a consumer, nonimplantable wearable device that can be worn on a wrist (e.g., a fitness wristband).

**Figure 5:**
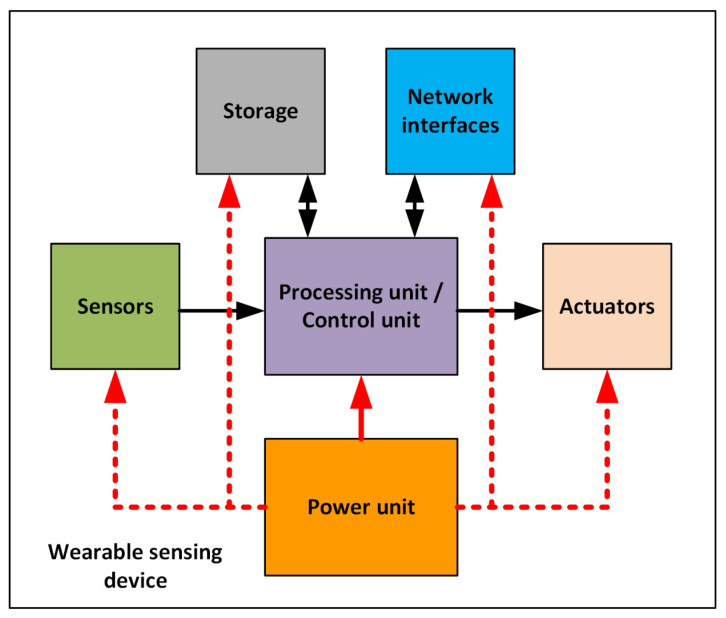
Typical components of a wearable sensing device. The red dotted line indicates possible connection.

**Figure 6:**
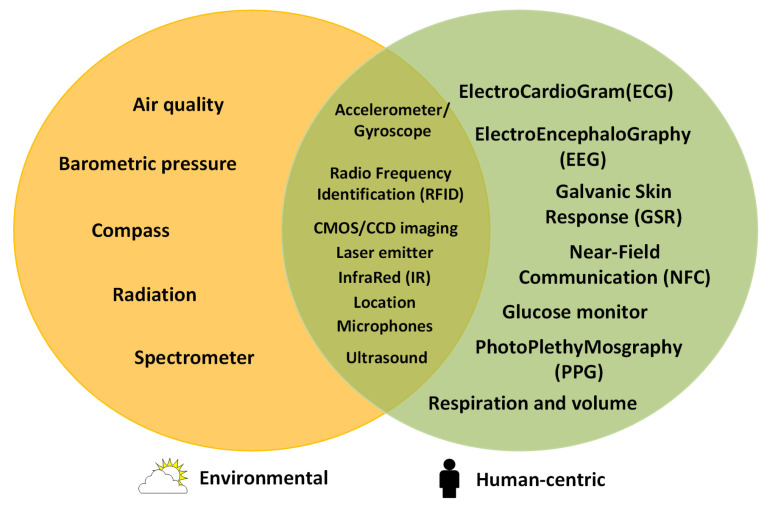
Typical sensors available in wearable devices grouped by type of collected data.

A wearable sensor device may be composed of the following components depending on its objectives and functionalities (as shown in Figure 5):

- A power unit. This component of the wearable sensor device provides the energy used by the wearable sensor device to operate. Some wearable devices may include rechargeable or nonrechargeable batteries and energy-harvesting technologies [43]. Table 2 presents different types of power-generating units that can be used.
- Sensors. These are electronic and microelectro-mechanical systems (MEMS) components that measure a physical quantity on the user (human-centric) or their surrounding environment (environmental). These sensors may be intrusive to the user (e.g., implanted in the body), with part of the wearable device worn by the user (e.g., smart fabrics [52] and photoplethysmography (PPG) sensors [53]), or carried around/worn by the user (e.g., location trackers [54]). Figure 6 shows a Venn diagram with wearable sensors grouped by type, and Table 3 describes each sensor.
- Processing/control unit. Based on the capabilities and/or design/objectives of the wearable, this component may perform basic calculations, filter data, or execute AI algorithms or control algorithms.
- Embedded storage media. Some wearable sensing devices have a flash-type storage media that stores sensor data for further analysis.
- Network interfaces. Using communication interfaces, a wearable sensor device may create a personal area network (PAN) with other wearable sensors, to communicate with a more powerful device such as a smart phone, or to directly forward data to a remote service.
- Actuators. Actuator components produce vibrations, sound, and visual cues (e.g., lights, screens, or heads-up displays) to notify the user about the device’s status. Some wearable sensing devices may not send data to a remote server/service, but they may provide automated feedback or execute an intrusive action on the user (e.g., an automatic defibrillator [55] and wearables for automated medication delivery [56,57] using microneedles) without the need for external systems, and some wearables provide information on a smartphone screen.

If smartphones are classified as external wearable sensing devices based on intrusiveness, as of 2021, the most-used wearable sensors (from those presented in Table 3) were the sensors embedded in most smartphones. These sensors are the microphone, location sensors, CMOS/CCD camera sensors, accelerometers, gyroscopes, and, to a lesser extent, the NFC interface as a contactless payment sensor. According to the 2021 Ericsson mobility report [58], as there are 5.5 billion smartphones in the world, there are 5.5 billion microphones, 5.5 billion location sensors, 5.5 billion CMOS/CCD cameras, and 5.5 billion accelerometers and gyroscopes collecting data in the world. If considered a wearable, the smartphone would be the most-used wearable during the COVID-19 pandemic caused by SARS-CoV-2. As these sensors are commonly available in most if not all smartphones, the NFC interface with 2.2 billion NFC-enabled smartphones/smartphone-like devices (e.g., tablets) [59] is next.

If the smartphone is not considered a wearable, then the most-available wearable sensors are accelerometers embedded in 708 million smartwatches and activity tracker units shipped between 2018 and 2021 (projection) [60]. However, if it is assumed that all true wireless stereo (TWS) hearables have microphones, then there would be 709 million microphones shipped as part of the TWSs sold between 2018 and 2021 (projection) [61]. After these sensors, the most commonly used sensor is the photoplethysmography(PPG) sensor available in many smartwatches, activity trackers, and pulse oximeters [62].

**Table 2:** Energy sources for wearable sensing devices.

Energy Source	Description	Examples of Wearable Sensing Devices
Nonrechargeable batteries	Use of standard-size small or specialized-size batteries that power a wearable sensing device	Insulin pumps, cochlear implants/devices, implantable cardioverter defibrillators
Rechargeable batteries	Lithium ion batteries that may be connected to an external power source to be recharged	Smart watches, smart phones, heart trackers, insulin pumps, digital stethoscopes [63], portable handheld ultrasound diagnostic devices [64]
Solar-powered	Use of photovoltaic (PV) cells to recharge a battery that powers a wearable	Smart bracelets [65], smart watches, external wearables such as tracking devices, smart fabrics
Radiofrequency (RF)	Use of antennas that extract energy from radio signals to recharge a battery or to power directly a wearable sensor	Radiofrequency identification (RFID) implants [66], bioelectronic stickers/tattoos [67]
Movement and mechanical waves	Use of piezoelectric devices to extract energy from human movements [68] or mechanical waves such as wind or ultrasound to recharge a battery or to power a device [69]	Implantable medical devices [69], wrist wearables [70]
Thermoelectric generators	Use of body heat to generate power to recharge a battery or to power directly a wearable sensor [71]	Biometric wearables and smart t-shirts for electrocardiogram monitoring [72]

**Table 3:** Sensor technologies for wearable sensing devices.

Sensor Type	Description/Application	Wearable Device Examples	Type of Collected Data
Smart fabrics (e-textiles)	Fabrics developed from traditional materials (e.g., cotton, polyester, nylon) combined with materials possessing electrical conductivity, or that can be embedded/uses to carry other sensors/electronic components. Some smart fabrics can detect the presence of chemical substances [73]	Zephyr compression shirt, Nadi X smart yoga pants	Human-centric
Electrocardiogram (ECG) sensor	Measures the electrical impulses of the heart muscle. Usually placed in contact with the skin. May be used in conjunction with implantable cardioverter defibrillators. Provides heart pulse data	Shimmer3 ECG chest unit, Apple Watch Series 6	Human-centric
Near-field communication (NFC)	Enables communication at short distances (less than 10 cm). Used as a wearable payment sensor [74,75]. Can be used to detect proximity and infer location, and for multiple-factor authentication methods [76].	NFC Ring, many smartphones, smartwaches	Human-centric
Galvanic skin response (GSR) sensor	Measures skin conductivity. Used in wearables to recognize stress levels/emotional state of an individual [77].	Empatica E4 wristband	Human-centric
Photoplethysmography (PPG) sensor	Measures blood volume changes. These sensors illuminate the skin of a wearer and measure light absorption to determine human body variables including heart rate [78,79], blood oxygenation levels [80], and blood pressure when used in conjunction with an ECG sensor [81].	Wellvue O_2_ Ring, pulse oximeters, most fitness bands and smart watches	Human-centric
Electroencephalography (EEG) sensors	Measure electrical activity in the scalp of a user. These devices can be used to diagnose abnormal brain activity when used in healthcare applications [82] or to control devices through brain–computer interfaces (BCIs) [83].	Emotiv EpocX	Human-centric
Glucose monitors	Monitor blood glucose levels for people with diabetes. Devices can monitor glucose levels continuously or at a single moment in time [84].	Dexcom G6 CGM	Human-centric
Infrared (IR) sensor	Measures skin or ambient temperature. Temperature can be used to predict ovulation in female mammals.	Ava fertility tracker	Human-centric/environmental
Accelerometer/gyroscope	Detects sudden accelerationmovement. Accelerometers can be used to detect and characterize human activities [85].	Shimmer3 IMU, Samsung Galaxy Watch 3, activity trackers, smartphones	Human-centric/ Environmental
Microphone	Detects sound. They can be used to detect health conditions, ambient sounds, activity, location contexts (e.g., being in a restaurant, hospital, home) [86].	Eko CORE family of stethoscopes/stethoscope attachments	Human-centric/Environmental
Location sensor	Tracks the locations/places where a user carrying a device with location may be [87]. Location sensors may be outdoor location or indoor location sensors and include technologies such as a global positioning system (GPS; United States), Galileo (European Space Agency), GLONASS (Russia), BeiDou (China) receivers, or the Navigation with Indian Constellation (India) systems. Indoor location technologies/sensors may include sonar-based, dead reckoning, Bluetooth low energy (BLE) beacons, among others [88].	Game Golf GPS receiver, Jiobit, Pet tracker, smartphones, most smartwatches	Human-centric/Environmental
Complementary Metal-Oxide Semiconductor (CMOS)/CCD imaging sensor	Takes photographs. When combined with AI, it may be used to detect objects and possibly recognize people’s identities without consent [89]. May be used to detect emotions in humans.	Iristick, Ray-Ban/Facebook Stories smart glasses, H1 head-mounted smart glasses, Microsoft HoloLens, Axon Body 2 body cameras, smartphones,	Human-centric/Environmental
Radiofrequency identification (RFID) tags	Store information about its wearer. RFID can be active or passive and can be used to track assets [90]. RFID can be used for location-based systems and to estimate crowd size in crowd-management systems [91].	3M RFID tags, ARDES Injection needle with RFID chip for cats and dogs, smartphones	Human-centric/Environmental
Laser emitter	Laser emitters are used to measure distances through light detection and ranging (LiDAR) and there are plans to be integrate them in future augmented reality (AR) glasses and smartphones [92]. A laser emitter can also be used for both acute and chronic pain management [93].	CuraviPlus Laser Therapy Belt for Lower Back Pain, future smart AR glasses and smartphones	Human-centric/Environmental
Ultrasound sensor	Detects objects in the proximity of a user/device [94]. Used also as an imaging sensor in handheld healthcare medical devices [64].	WeWALK smart cane, UltraCane, SonoQue, and Clarius portable handheld ultrasound devices	Human-centric/Environmental
Air quality sensor	Detects harmful gas concentrations/volatile components [95].	Atmotube PRO, TZOA, Flow 2 by plume labs	Environmental
Spectrometer	Separates and measures the spectral components reflected by a material. The light spectrum can be used to determine the components of the material [96].	GoyaLab IndiGo modular visible spectrometer	Environmental
Radiation sensor	Tracks ionizing [97] and nonionizing [98] radiation in the proximity of its wearer.	Instadose 2 Personal Radiation (X-ray) badge, Landauer RaySafe i3 Real-time Personal, Radiation Dosimetry, Landauee Tactical RadWatch	Environmental
Barometric pressure sensor	Detects barometric (atmospheric) pressure. Can be used to detect movement, activity [99,100], and altitude.	Garmin Fenix 5X	Environmental
Compass	Determines orientation and used for navigation	Most smartwatches	Environmental

## 3. Communication Technologies for Wearable Sensing

Advances in communication technologies support the current generation of wearable sensing services. From improvements in intrabody, body area networks (BANs), and personal area networks (PANs) to worldwide deployments of broadband wireless network connectivity, and computing paradigms such as cloud computing and blockchain, communication technologies are supporting many services that use wearable sensing technologies to deliver and provide value-added services to their users.

**Figure 7:**
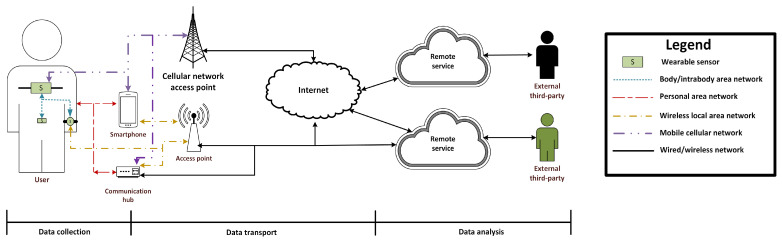
General architecture of wearable sensing systems.

Figure 7 shows a general architecture for a wearable sensing system. In this architecture, wearable sensor devices collect data and conduct filtering or execute basic data analysis and/or models trained using machine learning (ML) algorithms [23]. Some wearable sensing devices may connect to other sensors (intrabody area networks) or to a smartphone using BANs or PANs. At some point, and based on the design or features of the wearable sensing device, the latter may forward the data collected to a remote service using a cellular network or WLAN either directly connected to the Internet or via a smartphone or communication hub that serves as a gateway device for the wearable device. Depending of the application, specialized networks such as tactical communication networks and satellite communication may be used.

Today, technological advances in communication technologies are found in intrabody networks, BANs, and PANs. These networking technologies are used in wearable sensing to connect wearable sensor devices amongst themselves and to other devices such as a smartphone, a communication hub, or actuator devices over a short distance [101]. Wireless technologies used in these networks can be of two types: radiofrequency-based wireless body area network (RF-WBAN), and nonradiofrequency-based wireless body area networks (non-RF-WBAN). In the first group (RF-WBAN), technologies include Bluetooth and Bluetooth low energy [102,103], Zigbee [104], IEEE 802.15.6 WBAN [105], near-field communication (NFC) [106], as well as proprietary protocols such as Sensium [107] and ANT [108]. NFC is most used as a contactless payment sensor [109] over short distances (less than 10 cm). The drawbacks of RF protocols for wireless sensing devices and intrabody communications include radio signal degradation due to the composition of body tissues (signal attenuation) [110], broadcast of signals at low power to avoid damaging tissues due to heat dissipation, and power consumption issues related to continuous operation. In the second group (Non-RF-WBAN), the use of molecular communication [111,112,113], ultrasonic communication [114], and wired networks (e.g., the USB personal healthcare device) [115,116] have been proposed as alternatives to wireless communications for PANs and BANs.

Wireless sensing devices use wireless local area networks (WLANs) to connect to the Internet, to other sensing devices, or to local hubs that serve as gateways to other systems and networks using the Transmission Control Protocol/Internet Protocol (TCP/IP) network stack or dedicated protocols such as Continua [115] and ISO/IEEE 11073 [116]. While most wearables connect to WLANs, local hubs, or PANs (i.e., smartphones) to send data to remote services, newer wearables, especially in the consumer market, access remote services using mobile broadband [117].

Advances in mobile Internet broadband and cellular networks (4G/5G) along with decreasing costs have fueled the mass adoption and utilization of wearable sensing technologies and services. As of 2021 [58], there were more than 8 billion cellular subscriptions in the world. More than 80% of these subscriptions are mobile broadband connections and 5.5 billion are smartphone connections. According to Ericsson [58], part of the future improvements in network capacity provided by 5G cellular networks by 2024 will satisfy the growing demand for services including services making use of consumer wearable sensing devices. Other networking solutions exist for wearable sensing technologies in specific markets. These include Mobile Adhoc NETworks (MANETs), intranets, and satellite networks with proprietary protocols [118].

## 4. Remote Service Technologies for Wearable Sensing

In the previous section, we described computer network technologies enabling communication infrastructures for wearable sensing. While these technologies are responsible for transporting data between two entities (i.e., wearable sensors and a service provider), they do not implement a service on their own. The kind of data collected and why the data are being collected, used, and shared create value for stakeholders through the implementation of services. These services may fall into four major system categories [23]: location-based services (LBSs), human-centric sensing (HCS), participatory/crowdsensing systems (PS/CSs), and hybrids or combinations of these categories. Table 4 describes examples of these systems.

Wearable sensing services can be implemented using private servers [23], servers deployed in the cloud [119], and more recently as distributed apps (DApps) using blockchain and smart contract technology [120,121,122]. Remote service implementations making use of hybrid architectures between servers/the cloud and blockchain technology have recently been proposed for participatory/crowdsensing and human-centric systems [123,124,125,126]. A drawback of using public blockchains to store sensor data is the high monetary cost when data are uploaded to a public blockchain [127].

For services implemented using private servers and cloud services, remote services use technologies such as Structured Query Language (SQL) relational databases (e.g., Postgres, MySQL, MS SQL Server, and Oracle) and nonrelational (NoSQL) databases (e.g., Apache Ignite, Memcached, Cassandra, Hadoop/Hbase/HDFS, Azure Cosmos, Amazon S3, and Google Cloud Storage). Nonrelational databases are chosen by many of these services because they are useful for storing large amounts of data (big data) in real time. For example, data collected by Uber can be in the order of petabytes (PB) per day [128].

**Table 4:** Types of wearable sensing systems.

Type of System	Description	Examples of Systems
Location-based systems	Use location data to track, query, or provide a service based on location only [129].	Smart Caddie, OneBusAway [130], Jiobit pet tracking system, Uber, Lyft
Human-centric systems	Use sensors to monitor human-related physiological variables, activities and behaviors. Personal monitoring systems (e.g., fitness systems) and intelligent medical/healthcare systems fall into this category [131,132]	Fitbit Premium + Health, Garmin Connect, Samsung Health, Apple Healthkit
Participatory/ crowdsensing systems	Use collaborative data collected from a crowd to estimate communal parameters of interest [133,134] such as traffic, pollution, noise levels and others [135]. Participatory/crowdsensing systems include systems for crowd management [91,136,137], emergency management [138], and recently COVID-19 epidemiological systems based on mobile phones and sensors [10,11].	Crowdsync, COVIDNearby, CovidSens [139], MetroSense [140]
Hybrid systems	Systems making use of the characteristics of more than one class/kind of system above.	PokemonGo

Many of these systems are implemented in clouds using software engineering architectures based on microservices [141]. Microservices implement a remote service using a collection of small, independent services potentially deployed on different platforms or technology stacks [141]. Some of their advantages over monolithic architectures for software [142] include adaptability to changes in technology, reduced time-to-market (new features for a given remote service may be released as a microservice on its own), scalability, and flexible software engineering development practices (e.g., DevOps [143]) which suit many startup companies designing wearable systems and services. Data collected by remote systems can be analyzed using AI and ML techniques [26,45] such as deep learning (DL) [34], and feedback may be provided to users or to external third parties based on privacy policies or terms of use and commercial agreements [144].

## 5. Security and Privacy for Wearable Sensing

Security and privacy are two of the most significant aspects in the adoption of wearable sensing technology and systems in many application areas. For example, in mobile health (m-Health), if a wearable system that delivers medications or provides intrusive actions in the body of its user (e.g., implantable cardioverter defibrilators (ICD) with remote connectivity) has exploitable security flaws, the consequences could be catastrophic. From the privacy perspective, past research on smartphone-based sensing systems [25] demonstrated that certain kinds of data collected (e.g., location data) could be exploited to reveal aspects considered private for a user or a participant in a crowdsensing system. In this section, we first review security issues in wearable systems, and then review privacy aspects in wearables.

### 5.1. Security

Wearable sensing systems are susceptible to similar vulnerabilities and attacks found in other Internet of Thing (IoT) devices and systems. Security attacks, for wearable sensing technologies, can occur in a wearable sensing device, during data transport, or in the remote services collecting and analyzing data by exploiting vulnerabilities not considered at the design phase of a system. Table 5 summarizes vulnerabilities in wearable sensing devices.

In contrast to other IoT devices or computer systems that may be physically isolated or protected, the lack of physical security in wearable devices can be easily exploited by adversaries launching spoofing attacks to submit incorrect/fake data to a remote service [145,146]. A second type of spoofing attack (called mule attack [147]) may attempt to tamper with a wearable’s context or environment to make the wearable submit incorrect data [148,149]. For example, a mule attack on a fitness band or smartwatch to detect or register activity levels could be easily manipulated by an adversary by waving their arm or tying the wearable to a rope and make the fitness band or smartwatch rotate while standing at the same location [147]. Solutions proposed in the literature for spoofing attacks include the utilization of AI to recognize correct patterns/context [150] and the utilization of multiple sensors worn by a user to test the data collection context or to verify the data during collection phase [79,151,152,153].

When wearables are used in remote services collecting data from users to estimate environmental variables of interest (e.g., pollution, noise, or road traffic levels) through participatory or crowdsensing systems, spoofing attacks can be mitigated at the remote service by taking advantage of the redundant data collected by multiple users to estimate and filter out incorrect, erroneous, or fake data. Methods to filter out data in remote services include kriging, principal component analysis (PCA), Markov random fields (MRFs), Gaussian mixture models (GMMs), stochastic processes [154,155,156,157,158], and anomaly detection algorithms based on ML methods such as support vector machines (SVMs), neural networks (NNs) [159], and recent methods based on DL (i.e., convolutional neural networks (CNNs), and long short-term memory (LSTM) neural networks [160,161,162]).

A second vulnerability that can be exploited in wearable devices is the limited energy management and harvesting, which an adversary could exploit to perform battery exhaustion attacks and render a wearable ineffective in executing its tasks. In contrast to other IoT devices and computer systems, such as desktops, in which energy/power is not an issue because they are always connected to a reliable power source (i.e., a city’s power grid), most wearables use nonrechargeable or rechargeable batteries and/or energy-harvesting techniques (as we described in Section 1). Techniques available to mitigate this kind of attack include the development of power-aware frameworks and operating systems that continuously monitor the power consumption of the device [163,164], assessing the software’s power consumption before being implemented into a battery-powered device [165,166,167], and runtime anomaly detection methods that detect abnormal power patterns [168,169,170]. While most of these methods have been developed for smartphones, they can be adapted for wearables and used to detect and mitigate battery exhaustion attacks.

**Table 5:** Vulnerabilities in wearable sensing devices (adapted from [41]).

Vulnerability	Description	Examples of Attacks
Limited physical security	Unauthorized physical access to a wearable device by an adversary without difficulty	Physically damaging a device, spoofing attacks [145,146], manipulation of a device’s context/environment to make the device malfunction or incorrectly collect/register data [148,149]
Limited power	Wearable devices use batteries or energy harvesting techniques; attacks may drain their batteries and render them unusable	Battery exhaustion attacks [171]
Weak encryption	Use of encryption protocols that may not sufficiently protect data sent by a wearable due to energy limitations, processing power limitations, and bad software engineering practices	Eavesdropping, injection, and denial of service (DoS) attacks in health monitoring devices [172]
Weak authentication	Failure to authenticate a user, a wearable device, or data generated by a wearable due to energy, computational power, poor design, mode of use, or user interface constraints that may not allow the implementation of strong authentication protocols on a wearable device	Stealing, losing, or duplicating a physical token for a wearable device [173]
Unnecessary open ports	Devices may keep operating system (OS) ports/network addresses that may be exploited in security attacks or privacy violations	Tracking of users using botnets and Bluetooth low energy (BLE) [174]
Software vulnerabilities	Software may be implemented with errors or weak programming practices that make wearables vulnerable to security attacks; some of these weak practices include backdoors and errors during firmware updates	Attacks on fitness trackers during firmware updates [151] Logic bombs [175]

The use of encryption protocols that can be broken by an adversary with enough computational resources occurs when manufacturers use weak encryption in their devices (either in software and/or hardware). Weak encryption may enable attackers to eavesdrop on data in transit either to another wearable or to a remote service on the Internet. Weak encryption may occur due to limitations in the software, hardware, power availability, or weak programming practices, which may expose a wearable to attacks. In the past, research has shown that manufacturers sell consumer wearables with a lack of encryption, so can be attacked either through passively eavesdropping Bluetooth connections, through man in the middle (MITM) attacks, or by failing to encrypt data stored locally [172,176,177,178,179]. These attacks can violate user privacy, impersonate a user, or fabricate data submitted to a remote service. To mitigate this vulnerability, manufacturers should use hardware that supports strong encryption (e.g., ARM and Intel processors have hardware-specific extensions implementing Advance Encryption Standard (AES) algorithms), strong end-to-end encryption protocols (when supported by hardware/software),and good software engineering practices.

Weak authentication vulnerabilities arise when a wearable device, a wearable’s user, or data sent by a wearable cannot be authenticated due to due to energy, computational power, poor design, mode of use, or user interface constraints in the device or the system. Cryptographic authentication mechanisms available for other types of computers may not be enough to authenticate users, data, or wearable devices. For example, usually, login identifiers and passwords are used to authenticate a user in a laptop or desktop environment. However, because many wearable devices can be easily removed by users and worn by somebody else, the use of single-time authentication methods such as passwords are not enough to guarantee who is using a wearable device. To enforce authentication, various user authentication methods have been proposed based on biometrics [180], multifactor authentication mechanisms [76,181], and multiple wearable devices [182,183].

Unnecessary open ports make wearables susceptible to security and privacy attacks. While these attacks, so far, are less common than in other IoTs and computer systems, they are still present in wearables. For example, Issoufaly and Tournoux [174] highlighted how fingerprinting of wearables’ BLE medium access control (MAC) addresses can be easily exploited to track users via these addresses. In their research, they found that even though security and privacy features exist in BLE specifications, these features are rarely used. Robles-Cordero et al. [184] arrived at similar conclusions. Becker et al. [185] showed that even with BLE address randomization, passive tracking is possible. Knackmuß et al. [186] also explored unnecessary open port attacks, in which they used a packet sniffer tool to find an open port on a popular infusion pump. While open port vulnerabilities can be easily solved by having good product development practices, they pose a significant threat to wearables.

Other vulnerabilities of wearable devices that can be exploited are those arising from weak software development practices [187]. Software vulnerabilities can lead to malfunctioning, leaks in privacy, manipulation of data, or causing a wearable to execute attacks on other devices over a network (or the Internet). Software vulnerabilities may be caused by weak programming practices that can be present as part of the original software (firmware) with which a wearable is manufactured, or as part of firmware updates. Some of the vulnerabilities, such as inadequate encryption, inadequate authentication, and the unnecessary open ports previously discussed in this section, may be the result of weak programming practices. Weak programming practices can generate significant technical debt [188,189] in wearable sensing systems. Examples in the literature of possible attacks include remotely accessing implantable cardiac devices to make them fail [190] or to violate users’ privacy [191]. After Corbin [192] reported that former U.S. Vice President Dick Cheney’s pacemaker had software vulnerabilities that could enable hackers to cause heart attacks remotely, the former Vice President disabled the remote access capabilities of his pacemaker.

### 5.2. Privacy

Wearable sensing services may expose users to various privacy threats because the data collected by these services can be potentially linked back to users [193]. Research on user privacy perceptions on the utilization of consumer wearables has identified privacy concerns related to social implications, criminal abuse, facial recognition, access control, social media sync, right to forget, surveillance and sousveillance, speech disclosure, and surreptitious audio/video (A/V) recordings when using a device, which may continuously register users’ actions [194]. Table 6 summarizes these privacy concerns.

These concerns can be classified into three main privacy issues categories, which include context privacy, bystanders’ privacy, and external data sharing privacy [38]. Table 7 presents each privacy concern with a privacy issue and the related solutions found in the literature.

The first privacy issue we present is context privacy. Context privacy comprises the context/actions deemed private by a wearable user that can be inferred based on the data or metadata collected through a wearable. Many wearables are used continuously, and users may not remember that the utilization of a wearable may possibly register all users’ actions (and data about users’ surroundings), and private information could be inferred [195]. Solutions to solve this issue include methods that only collect data when the user desires it [196,197,198], and methods to avoid or deny data collection when the user wishes [199]. For both types of solutions, the idea is for the user to create rules at different levels that may be based on raw sensor data readings, or more complex rules that may involve activity recognition at the wearable device.

**Table 7:** Privacy concerns and issues for wearables (adapted from [38]).

User Privacy Concern	Privacy Issue	Recently Proposed Solutions
Access control Location disclosure Social implications Discrete display and visual occlusion User’s fear Speech disclosure Right to forget	Context privacy	Virtual trip lines [196] Bubble sensing [197] Privacy bubbles [198] Virtual walls [199]
Speech disclosure Social implications Facial recognition (identification) Surreptitious A/V recording User’s fears Location disclosure	Bystanders’ privacy	BlindSpot [200] Using IR to disable devices [201] Using Bluetooth to disable capturing device [202] Virtual Walls [199] Privacy-aware restricted areas [203] PrivacyEye [204] PrivacyVisor [205] PrivacyVisor III [206] Perturbed eyeglass frames [207] Respectful cameras [208] Negative face blurring [209] FacePET [210] I-Pic [211]
Access control Location disclosure Social implications User’s fear Criminal abuse Social media sync Discrete display and visual occlusion Right to forget	External data-sharing privacy	k-anonymity [212] l-diversity [213] t-closeness [214] Differential privacy [215,216] Homomorphic encryption [217,218]

A second privacy issue with wearables is bystanders’ privacy. Bystanders’ privacy is the issue of the re-identification of third parties (bystanders) who have not provided consent when a sensing device is used in a bystander’s surroundings [89]. Research in the area of bystanders’ privacy has focused on understanding user and bystander privacy perceptions and privacy norms on the utilization of camera-enabled and voice-capturing devices in shared spaces [194,204,208,219,220,221,222,223,224,225,226,227] and on the development of systems for bystander privacy protection [200,201,202,203,204,205,206,207,208,209,210,211].

In the area of privacy perceptions and norms of camera-enabled and voice-capturing devices in shared spaces, past research has identified a conflict in spaces shared between users and bystanders [219,224,225,228], a desire of bystanders to have some control over what may be recorded and shared about them [220,221,222], and the meaning and definition of the contexts that affect the social meaning of privacy [223,228]. To protect bystanders’ privacy, research has focused on policies and systems to disable devices [200,201,202,203,204] and obfuscation of faces in photos in some systems [205,206,207,208,209,210,211].

Another privacy issue with wearables is external data sharing. When data are collected by remote services (such as systems described in Table 4), the issue of external data-sharing relates to how a remote service can protect a wearable’s user privacy when shared with a third party. Third-party data-sharing can occur because of commercial agreements by which an external party provides some type of service on behalf of the remote service provider, or because the remote service provider sells the collected sensor data. Solutions that address this issue to protect the privacy of the collected data focus on anonymization methods in databases and cryptographic-based methods.

Anonymization methods in databases protect wearable users’ privacy by providing meaningful third-party data (in the statistical sense) without releasing data that can identify users. Depending on the kind of data to be released, these methods can be classified as methods for microdata release or methods for statistical data release [229]. In the first group (methods for microdata release), the goal is to protect users’ private data by de-identifying private attributes of identities during the release of microdata (i.e., records from a database). Examples of methods for microdata release include k-anonymity [212], l-diversity [213], and t-closeness [214]. In the second group (methods for statistical data release), we protect user privacy by guaranteeing that the release or calculation of a statistical result (such as an average) using collected data cannot reveal information about the specific records or a user. An example of a method that falls into this latter category is differential privacy [215,216,230]. A third class of methods available to protect data when externally shared is cryptographic-based methods, using homomorphic encryption techniques [217,218]. Homomorphic encryption allows a remote service provider to release data encrypted so that an external party can perform calculations on the encrypted data without revealing the original data or identifiable data.

## 6. Challenges and Research Opportunities for Wearable Sensing Technologies

In the previous sections, we reviewed the technological advances in various aspects of wearable sensing systems. Although significant work has been untertaken on wearable sensing systems in the last ten years, in this section, we identify areas of interest that need further research. These areas include security, privacy, 6G and ML at the edge (federated learning), energy harvesting and management, and interoperability.

### 6.1. Security

Even though we reviewed the vulnerabilities of and possible solutions for wearable technologies, security will continue to play an important role in the research and development and use of and trust in wearable devices. Attacks on wearables devices launched by an adversary can have catastrophic consequences for a user, especially if the wearable device is used in m-Health systems [158,231]. In addition, wearables connected to the Internet could be hacked and used to attack other systems. This creates the issue of developing wearables with a security-by-design paradigm [232,233] to identify security risks and vulnerabilities during the design and development phases of a system rather than mitigating them after cyberattacks. The Mirai botnet illustrates this issue.

During late 2016, a distributed denial of service (DDoS) was launched over the Internet using a botnet called Mirai, which infected approximately 65,000 IoT devices such as DVR devices, IP cameras, routers, and printers [234]. Mirai converted them into zombies (i.e., devices controlled by a remote machine) and then used the infected IoT devices to launch various DDoS attacks on DNS servers. According to Antonakakis et al. [234], the attack was enabled by the design decisions of a small group of consumer electronics manufacturers. Although this attack used nonwearable IoTs, it underscores the need to incorporate security as part of the design process of a wearable. More research is needed to continue protecting current and next-generation wearables and mitigate emerging threats.

### 6.2. Privacy

One of the privacy challenges for users of wearable sensing technologies is making informed choices about the sensing devices that consumers buy and use. This highlights the importance of the development of usable privacy policies [235]. Privacy policies disclose the practices of remote services in terms of aspects such as data collection, management, and sharing about websites, services, and devices that consumers buy and use [144].

Although laws regarding the requirements of privacy policies and their content may differ from country to country, the General Data Protection Regulation (GDPR) regulation standardized these requirements for the European Union (EU) countries by mandating remote services to provide a privacy policy if these services control and process the personal data of individuals located in the EU, independent of the services being physically located in the EU or not [236]. However, companies whose markets may not be EU countries are not bound by this requirement, which may leave users in non-EU countries without an understanding of how remote services use with the data they collect through wearables. Furthermore, if privacy policies are provided, the accessibility and understandability of these privacy policies remain challenges. More research is needed on usable and practical privacy policies for wearables and for general IoT devices.

Another open privacy challenge for wearable sensing technologies is the control of users’ sensor data collected by remote services and how data are shared with third parties (i.e., external systems or services) without users’ consent. There are two aspects to this issue:the first is how the users know that their data are being shared with third parties (an issue related to privacy policies) and when this occurs; the second is how users’ privacy may be protected when data are externally shared. While we reviewed solutions for the second issue, more work is needed to enable data-sharing with third parties while protecting users’ privacy and allowing users to have control over their data.

Finally, bystanders’ privacy [89] continues to be an open challenge in wearable devices. Even though we reviewed some solutions to address this issue, more research is needed to protect bystanders and to develop wearables with a focus on facial and voice privacy protection.

### 6.3. 6G and Machine Learning at the Edge (Federated Learning)

Currently, 5G networks continue to be deployed worldwide to support increasing mobile Internet traffic, including a growing demand for wearable sensing services [58]. However, the next generation of cellular networks (6G) will be designed to meet the demands of an intelligent, fully connected digital world [237,238]. It is expected that 6G networks will support pervasive and ubiquitous sensing services under very-low-latency requirements in the order of hundreds of microseconds [237]. To accomplish this, research is needed to integrate AI to dynamically predict traffic requirements, conduct adaptive and intelligent network management, and enhance remote sensing services.

Currently, remote services collect and store large amounts of wearable sensor data in clouds using machine learning (ML) and deep learning (DL) techniques to extract knowledge. Advances in graphical processing units (GPUs) have fueled a revolution in deep neural networks (DNNs) and DL by shortening the training time of DNN/DL models from months to hours. However, 6G networks will require part of this process to be conducted at edge devices (e.g., wearables, cellular phones, IoTs).

Federated learning (FL) [239] is a paradigm in ML/DL that is being investigated to train AI models using a decentralized process that is executed at edge devices. As ML/DL models in FL are trained at edge devices that collaborate among themselves rather than in centralized servers in a cloud (or data centers connected to the core of the Internet), less data are sent over the Internet, with advantages such as improved privacy, security, and access rights management. The resulting models built using FL can be as robust as models built using centralized ML/DL and are useful in areas such as telecommunications [240] and healthcare [241], amongst others. Additionally, research on techniques against adversarial ML, wherein wrong or fake data are used on purpose to make AI models fail, in FL environments is needed.

### 6.4. Energy Harvesting and Management

Advances in software, hardware, communication, computation, sensor technology, and AI have enabled the current generation of wearables; their power consumption will become increasingly important in the future. This will make energy harvesting and management one of the most important aspects that will drive the design and implementation of the next generation of wearables. Without power, a device cannot work, and one of the goals of using wearables is that a wearable device can continue performing its tasks without having to recharge, or if it needs to be recharged, it can be achieved in a way that is not cumbersome for its user (e.g., using wireless charging).

To improve energy management in wearables, hardware research should focus on the optimization of specific tasks of the wearable without relying on software. In particular, given the use of DL to classify and recognize wearable sensor data and the future use of FL to train ML models at the edge, hardware research can focus on alternate implementations of deep neural networks and other ML models to minimize power. Currently, FL algorithms may use high-power-consuming GPUs in battery-powered devices such as wearables to train distributed DL models, which may affect the usefulness of a wearable device [242,243]. An example of hardware-based optimization for DL models is the IBM Fusion chip [244], which can encode an artificial neural network analogically using phase-change memory (PCM) circuits performing classification without using a microprocessor, thus accelerating a classification task while minimizing power. Other areas of research include intelligent software management to predict power usage and to switch among different power profiles (software optimizations such as decreasing the clock rate frequency [43]) and harvesting techniques that can collect energy from a wearable’s environment to enable its continuous operation.

A second aspect of energy management is the cost of energy needed to enable a wearable sensing system, from fabrication to operation and retirement or disposal. According to Smil [245], and using a concept called embodied energy (the sum of energy required to produce a good or service [246]), an approximation to the embodied energy of the production of all laptops, tablet, and mobile phones sold in 2015 was 1 EJ (1 × 10^18^ Joules) with an approximate weight of 550,000 metric tons; the 72 million cars sold during the same year accounted for 7 EJ of energy, while weighting about 100 million tons. According to his analysis, while the cars weighed 180 more times that of all portable electronics, they required only seven times as much energy to make [245]; while a car may last for a decade (or longer), many portable devices are disposed only after two years of operation. Cellular networks, which are needed for many future wearables, face a similar issue. In a research study, Humar et al. [247] found that embodied energy in cellular networks cannot be neglected because it accounts for an important and significant amount of their total energy consumption, and that embodied energy should be seriously considered in the design and development of other devices and systems used in the telecommunications sector (e.g., data centers). Thus, more work is needed to better estimate, optimize, and manage the energy consumption of telecommunication systems (such as wearable sensing systems) across their full lifecycle.

### 6.5. Interoperability

Wearable payment systems, fitness, and medical wearables are three of the fastest-growing market segments of wearable services. While the financial payments industry has worked (since the early years of 2000) to make payment systems interoperable and secure through standards such as the Payment Application Data Security Standard (PA-DSS) [16,248,249], this was not the case for fitness wearable services as of 2021.

On the fitness wearables side, the competition among many start-ups and well-established technological companies to position themselves as major players in the wearables market has made their wearable ecosystems closed ecosystems, with the exception of when a wearable/tech company takes over another wearable company (e.g., Google’s Fitbit acquisition in 2021 for USD 2.1 billion [250]), or when a company partners through business deals with third parties, including insurance companies or employee wellness benefit services. For example, Virgin Pulse, as of October of 2021, was supporting eight wearable brands including Fitbit, Misfit, Garmin, Polar, Withings, MiBand (via VP Mobile App), Apple Watch (via VP Mobile App), and the Samsung Gear family (via VP Mobile App) [251]. Thus, which data can be shared among different systems is left to each company, which may hinder interoperability. On the medical wearables side (m-Health), the regulations to determine if a wearable is an approved medical device and what constitutes an electronic health record (EHR) varies from country to country [252,253]. For example, in the U.S., the process to approve a device for the purpose of medical diagnosis, cure, mitigation, and treatment of disease in humans or other animals is managed through the U.S. Food and Drug Administration (FDA/USFDA) [254]. Depending on the intrusiveness and risks to the human or animal, the device can fall into three classes (I being the lowest risk and III being the highest risk) and can take up approximately eight months to be FDA-approved. However, it can take much more time to document all the needed information for safety approval. For a wearable with connectivity (wired, wireless, or public Internet or intranet), the FDA submission should include a cybersecurity review of the device similar to the one described on the guidance document titled *Content of Premarket Submissions for Management of Cybersecurity in Medical Devices* [255]. A submitting organization can use a list of FDA-recognized cybersecurity consensus standards for IT and medical device security to document cybersecurity management. Table 8 presents these FDA-recognized standards (as of October 2021).

For EHR records in the U.S., if the data records generated by a medical device will be stored as part of an electronic health record (EHR), then different guidelines are used, which include the Health Insurance Portability and Accountability Act (HIPAA) and the Health Information Technology for Economic and Clinical Health (HITECH) Act. The largest companies in terms of market share developing software for EHR management tend to have closed networks (allowing sharing of EHRs easily only amongst network members of a specific company [256]), which has prompted the creation of alliances to share EHRs among smaller EHR software providers and practitioners (e.g., CommonWell Health Alliance [257]), lately as part of the U.S. Coronavirus Aid, Relief, and Economic Security Act (U.S. CARES Act) [258], giving hospitals and medical practitioners the option to use application programming interfaces (APIs) to exchange data using the recently developed United States Core Data for Interoperability (USCDI), which is a standardized set of health data classes and elements to allow interoperable exchange of health information nationwide. The second version of the USCDI was released in July 2021 [259].

While it is not mandated for fitness wearables companies to use medical-level standards, the latest development of standards for healthcare devices can provide interoperability among consumer wearables services in a secure and privacy-protected way, which may benefit users in the near future.

## 7. Conclusions

Over the last few decades, we have witnessed significant developments in the design and deployment of wearable sensing technologies. The size and cost of these technologies continue to decrease while their performance and capabilities continue to improve, making them increasingly pervasive in a wide range of applications. We foresee that wearable sensing technologies and services will continue to be improved and deployed worldwide in the future. In this work, we reviewed technological advances that have made wearable sensing possible and affordable. We described different types of wearable sensors, communication and remote services technologies, and security and privacy issues related to wearable devices. We also reviewed the use of consumer wearables during the COVID-19 pandemic caused by SARS-CoV-2. Finally, we discussed research challenges that must be addressed to further improve wearable sensing systems in terms of their designs, energy consumption, security, privacy, and interoperability.

In this review, we did not discuss the extensive use of different types of sensors that track human behaviors (e.g., movements of elderly people or flexing a finger) for different types of users in various types of environments, the safety of wearable sensors (we only presented a summary of FDA-approved standards), integration issues with body area networks (BANs) with other emerging technologies (e.g., fog, edge, and 6G), or AI-enabled sensors that could play a pivotal role in future medical services. In the future, we will explore some of these issues.

## Figures and Tables

**Table 1:** Summary of survey works in mobile and wearable sensing.

References	Year	Title	Remarks
[24]	2010	*A survey of mobile phone sensing*	Review of applications and architectures for smartphone sensing in human-centric and participatory sensing systems. No mention of wearables
[25]	2011	*A survey on privacy in mobile participatory sensing applications*	Review of privacy mechanisms for smartphone-based crowdsensing systems. No mention of wearables.
[26]	2012	*A survey on human activity recognition using wearable sensors*	Review of machine learning (ML) models to classify activities using wearables. Review does not include deep learning (DL) systems.
[27]	2012	*Mobile phone sensing systems: A survey*	Review of mobile-smartphone-based sensing applications in participatory/crowdsensing settings. Mentions two systems that, as of 2012,used electrocardiogram (ECG) sensors.
[28]	2013	*Mobile sensing systems*	Review of mobile sensing systems based on smartphones and their communication architectures. Provides short review on security.
[29]	2014	*Wearables: Fundamentals, advancements, and a roadmap for the future*	Review of wearable technology as of 2014 with a focus on sensors and applications. Does not review security or privacy issues.
[30]	2015	*A survey of incentive techniques for mobile crowd sensing*	Review of monetary and nonmonetary incentives mechanisms for mobile crowdsensing systems based on smartphones. Incentives are important in crowdsensing to recruit participants to collect data.
[31]	2015	*A survey on energy-aware security mechanisms*	Reviews energy-aware security mechanisms for WSNs, mobile devices (focus on smartphones), and network nodes as of 2015. Review does not mention wearables.
[32]	2016	*Pervasive eHealth services a security and privacy risk awareness survey*	Presents risk awareness and perception for eHealth wearables using Amazon Mechanical Turk.
[33]	2016	*Incentive mechanisms for participatory sensing: Survey and research challenges*	Review of application-specific and general-purpose incentive mechanisms for mobile crowdsensing systems based on smartphones.
[34]	2016	*Deep, convolutional, and recurrent models for human activity recognition using wearables*	Reviews and evaluates of deep learning methods for human activity recognition.
[35]	2017	*A survey of wearable devices and challenges*	Review focuses on consumer wearables available as of 2017. Work also addresses security, power, task offloading, and machine learning. Work does not address privacy issues.
[36]	2017	*The use of wearables in healthcare–challenges and opportunities*	Reviews applications of wearables in healthcare from the application perspective.
[37]	2017	*A survey on smart wearables in the application of fitness*	Review of wearables available as of 2017 in the context of fitness. Work does not address security, privacy, power, or ML in wearable systems.
[17]	2017	*Mobile payment systems: secure network architectures and protocols*	Describes architectures and protocols to enable mobile payments. From the device perspective, it focuses on mobile phones. No mention of wearables.
[38]	2018	*Privacy issues and solutions for consumer wearables*	Review of privacy issues in consumer wearables. Work does not address power or machine learning.
[39]	2018	*A critical review of consumer wearables, mobile applications, and equipment for providing biofeedback, monitoring stress, and sleep in physically active populations*	Review of the utilization of consumer wearables for stress and sleep monitoring. No privacy or security issues mentioned in the paper.
[40]	2018	*Wearables and the medical revolution*	Reviews the utilization of wearables for medical use (m-Health). No privacy or security issues reviewed in the paper.
[41]	2019	*Demystifying IoT security: An exhaustive survey on IoT vulnerabilities and a first empirical look on Internet-scale IoT exploitations*	Review of security issues and solutions in Internet of Things (IoT) systems. Review does not mention wearables.
[42]	2019	*Buddy’s wearable is not your buddy: Privacy implications of pet wearables*	Review of privacy issues and possible privacy violations or privacy leakages to owners of pets (pet parents) by having their pets use wearables.
[43]	2020	*Design architectures for energy harvesting in the Internet of Things*	Reviews power and energy harvesting techniques for Internet of Things (IoT) devices including wearable devices.
[44]	2020	*A comprehensive overview of smart wearables: The state of the art literature, recent advances, and future challenges*	Bibliographic review of published works related to wearable devices. This work reviews published works from 2010 to 2019 (before the COVID-19 pandemic).
[45]	2020	*Use of wearable sensor technology in gait, balance, and range of motion analysis*	Review of wearables and ML systems with a focus on gait analysis.
[46]	2020	*Wearables and the Internet of Things (IoT), applications, opportunities, and challenges: A Survey*	Review of sensors and applications of wearables before the COVID-19 pandemic. This work does not review security, privacy, or ML.
[9]	2020	*A survey of COVID-19 contact tracing apps*	Reviews contact tracing apps developed during the COVID-19 pandemic.
[47]	2021	*Wearables for Industrial Work Safety: A Survey*	Review of wearables in the context of industrial settings. Work focuses on applications of wearables for industry.
[48]	2021	*A survey on wearable technology: History, state-of-the-art and current challenges*	Review provides a comprehensive historical review of wearables devices. Reports on applications and some aspects of security and privacy.

**Table 6:** Wearable user’s privacy concerns, as researched by the authors of  [194].

Privacy Concern	Description
Social implications	Unawareness by a network of friends regarding data being collected about them
Criminal abuse	Fear that wearable data will be used by criminals to harass a user
Facial recognition	Association and recognition of a bystander to a place or a situation where the bystander would not wish to be recognized by others
Access control	Fear of users of third-party service providers sharing data without consent
Social media sync	Immediate publishing or sharing by the wearable device without the knowledge of the user
Discrete display and visual occlusion	Notifications/information of users that might be seen by bystanders who should not have access
*Right to forget*	The user’s wish to delete collected data that he or she wants to forget
User fears: surveillance and sousveillance	Continuous tracking of user activities that might make the user feel that no matter what they do, everything is recorded
Speech disclosure	Capturing speech that a user or bystanders would not want to record or share
Surreptitious A/V recording	Recording of video without permission that might affect bystanders
Location disclosure	Fear of sharing a location inadvertently to third parties that should not have access

**Table 8:** U.S. FDA-recognized standards for medical informatics security.

FDA Date	FDA Number	Organization	Organization Designation/Date	Standard
7 June 2021	13-119	ANSI ISA	62443-4-1-2018	Security for industrial automation and control systems Part 4-1: Product security development life-cycle requirements.
7 June 2021	13-118	IEEE	Std 11073-40102:2020	Health informatics-Device interoperability. Part 40102: Foundational-Cybersecurity-Capabilities for mitigation.
7 June 2021	13-117	IEEE	Std 11073-40101-2020	Health informatics-Device interoperability Part 40101: Foundational-Cybersecurity-Processes for vulnerability assessment.
6 July 2020	13-115	IEC IEEE ISO	29119-1 First edition 2013-09-01	Software and systems engineering-Software testing-Part 1: Concepts and definitions
6 July 2020	13-114	IEEE	Std 11073-10101-2019	Health informatics-Point-of-care medical device communication. Part 10101: Nomenclature
23 December 2019	13-112	AAMI	TIR97:2019	Principles for medical device security-Postmarket risk management for device manufacturers
15 July 2019	13-109	AAMI ANSI UL	2800-1: 2019	(American National Standard) Standard for Safety for Medical Device Interoperability
7 June 2018	13-104	ANSI UL	2900-2-1 First Edition 2017	Standard for Safety Software Cybersecurity for Network-Connectable Products Part 2-1: Particular Requirements for Network Connectable Components of Healthcare and Wellness Systems
4 December 2017	13-103	IEC	TR 80001-2-9 Edition 1.0 2017-01	Application of risk management for IT-networks incorporating medical devices-Part 2-9: Application guidance-guidance for use of security assurance cases to demonstrate confidence in IEC TR 80001-2-2 security capabilities
4 December 2017	13-102	IEC	TR 80001-2-8 Edition 1.0 2016-05	Application of risk management for IT-networks incorporating medical devices-Part 2-8: Application guidance-guidance on standards for establishing the security capabilities identified in IEC TR 80001-2-2
21 August 2017	13-97	IEC	82304-1 Edition 1.0 2016-10	Health software-Part 1: General requirements for product safety
21 August 2017	13-96	ANSI UL	2900-1 First Edition 2017	Standard for Safety Standard for Software Cybersecurity Network-Connectable Products Part 1: General Requirements
23 December 2016	13-85	CLSI	AUTO11-A2	Information Technology Security of In Vitro Diagnostic Instruments and Software Systems; Approved Standard-Second Edition
27 June 2016	13-83	AAMI	TIR57:2016	Principles for medical device security-Risk management.
14 August 2015	13-78	IEC ISO	30111 First edition 2013-11-01	Information technology-Security techniques-Vulnerability handling processes
14 August 2015	13-77	IEC ISO	29147 First edition 2014-02-15	Information technology-Security techniques-Vulnerability disclosure
27 January 2015	13-70	IEC	TR 80001-2-5 Edition 1.0 2014-12	Application of risk management for IT-networks incorporating medical devices-Part 2-5: Application guidance-Guidance on distributed alarm systems
6 August 2013	13-62	IEC	TR 62443-3-1 Edition 1.0 2009-07	Industrial communication networks-Network and system security-Part 3-1: Security technologies for industrial automation and control systems
6 August 2013	13-61	IEC	62443-2-1 Edition 1.0 2010-11	Industrial communication networks-Network and system security-Part 2-1: Establishing an industrial automation and control system security program
6 August 2013	13-60	IEC	TS 62443-1-1 Edition 1.0 2009-07	Industrial communication networks-Network and system security-Part 1-1: Terminology concepts and models
6 August 2013	13-44	IEC	TR 80001-2-3 Edition 1.0 2012-07	Application of risk management for IT Networks incorporating medical devices-Part 2-3: Guidance for wireless networks
6 August 2013	13-42	IEC	TR 80001-2-2 Edition 1.0 2012-07	Application of risk management for IT Networks incorporating medical devices-Part 2-2: Guidance for the disclosure and communication of medical device security needs risks and controls
6 August 2013	13-38	IEC	80001-1 Edition 1.0 2010-10	Application of risk management for IT-networks incorporating medical devices-Part 1: Roles responsibilities and activities

## Abbreviations

The following abbreviations are used in this manuscript:

cm	centimeter
4G	Fourth-generation cellular networks
5G	Fifth-generation cellular networks
6G	Sixth-generation cellular network
AAMI	Association for the Advancement of Medical Instrumentation
API	Application Programming Interface
A/V	Audio/Video
AES	Advanced Encryption Standard
AI	Artificial Intelligence
ANSI	American National Standards Institute
AR	Augmented Reality
BAN	Body Area Network
BCI	Brain-Computer Interfaces
BLE	Bluetooth Low Energy
CARES	U.S. Coronavirus Aid, Relief, and Economic Security Act
CCD	Charged Coupled Device
CLSI	Clinical & Laboratory Standards Institute
CMOS	Complementary Metal-Oxide Semiconductor
CNN	Convolutional Neural Network
COVID-19	Coronavirus Disease 2019
DApps	Distributed Apps
DDoS	Distributed Denial of Service
DL	Deep Learning
DNN	Deep Neural Network
DNS	Domain Name System
DoS	Denial of Service
DVR	Digital Video Recorder
ECG	Electrocardiography
EEG	Electroencephalography
EHR	Electronic Health Record
EJ	Exajoule
EU	European Union
FDA	Food and Drug Administration
FL	Federated Learning
GDPR	General Data Protection Regulation
GLONASS	Global Navigation Satellite System
GMM	Gaussian Mixture Model
GPS	Global Positioning System
GPU	Graphical Processing Unit
GSR	Galvanic Skin Response
HCS	Human-Centric Sensing
HDFS	Hadoop Distributed File System
HIPAA	Health Insurance Portability and Accountability Ac
HITECH	Health Information Technology for Economic and Clinical Health
IEC	International Electrotechnical Commission
IEEE	Institute of Electrical and Electronics Engineers
IoT	Internet of Things
IT	Information Technology
IR	InfraRed
ISA	International Society of Automation
ISO	International Organization for Standardization
LAN	Local Area Network
LBS	Location-Based Services
LiDAR	Light Detection And Ranging
LSTM	Long Short-Term Memory
MAC	Medium Access Control
MANET	Mobile Adhoc NETworks
MEMS	Microelectromechanical Systems
m-Health	Mobile Health
MITM	Man In the Middle
ML	Machine Learning
MRF	Markov Random Field
NFC	Near-Field Communication
NN	Neural Network
OS	Operating System
PA-DSS	Payment Application Data Security Standard
PAN	Personal Area Network
PB	Petabytes
PCA	Principal Component Analysis
PCM	Phase-Change Memory
PPG	Photoplethysmography
PS/CS	Participatory/Crowdsensing Systems
PV	Photovoltaic
RF	Radiofrequency
RFID	Radiofrequency Identification
SARS-CoV-2	Severe Acute Respiratory Syndrome Coronavirus 2
SQL	Structured Query Language
SVM	Support Vector Machine
TCP/IP	Transmission Control Protocol/Internet Protocol
TWS	True Wireless Stereo
UL	UL Incorporated, previously known as Underwriters Laboratories
USD	US Dollars
USFDA	US Food and Drug Administration
WLAN	Wireless Local Area Network
WSN	Wireless Sensor Network
WWAN	Wireless Wide Area Network
